# Associations between dietary total antioxidant capacity and sarcopenia: a cross-sectional study

**DOI:** 10.1186/s12937-024-00933-5

**Published:** 2024-07-31

**Authors:** Azadeh Aminianfar, Rezvan Hashemi, Fatemeh Emami, Ramin Heshmat, Ahmadreza Dorosty Motlagh, Ahmad Esmaillzadeh

**Affiliations:** 1 Students’ scientific Research Center, Tehran University of Medical Sciences, Tehran, Iran; 2 Research Center for Biochemistry and Nutrition in Metabolic Diseases, Kashan University of Medical Sciences, Kashan, Iran; 3 Department of Geriatric Medicine, Ziaeian Hospital, Tehran University of Medical Sciences, Tehran, Iran; 4 Department of Community Nutrition, Isfahan University of Medical Sciences, Isfahan, Iran; 5 Ebne Sina Hospital, Iran University of Medical Sciences, Tehran, Iran; 6 Obesity and Eating Habits Research Center, Endocrinology and Metabolism Molecular - Cellular Sciences Institute, Tehran University of Medical Sciences, Tehran, Iran; 7 Department of Community Nutrition, School of Nutritional Sciences and Dietetics, Tehran University of Medical Sciences, P.O. Box 14155-6117, Tehran, Iran; 8 Chronic Diseases Research Center (CDRC), Endocrinology and Metabolism Population Sciences Institute, Tehran University of Medical Sciences, Tehran, Iran

**Keywords:** Sarcopenia, Total antioxidant capacity, d-TAC, Elderly, Cross-sectional

## Abstract

**Background:**

No study has investigated the relationship between dietary total antioxidant capacity and sarcopenia so far.

**Objective:**

This study aimed to investigate the association between dietary Total Antioxidant Capacity (d-TAC) and sarcopenia in elderly adults.

**Methods:**

In this cross-sectional study we enrolled 300 elderly people (150 men and 150 women) aged ≥ 55 years using cluster random sampling method. Sarcopenia was defined based on European Working Group on Sarcopenia (EWGSOP) definition. A DXA scanner, a squeeze bulb dynamometer and a 4-Meter walk gait speed test was used to measure Appendicular Skeletal Muscle (ASM), muscle strength and muscle performance respectively. We also used a Block-format 117-item food frequency questionnaire (FFQ) to assess dietary intakes of participants. Multivariable logistic regression models were applied to examine the association between d-TAC and sarcopenia.

**Results:**

Mean ± SD age of study participants and their BMI was 66.8 ± 7.72 year and 27.3 ± 4.2 kg/m^2^, respectively. People in the highest tertile of d-TAC had the greatest hand grip strength (11.9 ± 3.63 vs. 10.4 ± 3.55 psi, *p* = 0.009) and had lower odds of sarcopenia compared with those in the lowest tertile, either before (OR = 0.39; 95% CI: 0.17, 0.88) or after adjustment for potential confounders (OR = 0.33; 95% CI: 0.11, 0.95). No other significant association was seen between d-TAC and components of sarcopenia.

**Conclusion:**

We found an inverse association between dietary total antioxidant capacity and odds of sarcopenia. No significant association was seen between d-TAC and individual components of sarcopenia. Further studies are needed to confirm our findings.

**Supplementary Information:**

The online version contains supplementary material available at 10.1186/s12937-024-00933-5.

## Introduction

Sarcopenia, a serious public health problem, is characterized by low muscle mass and strength and physical function. It is a degenerative syndrome occurs during aging which influences old people both physiologically and psychologically [1, 2]. The global prevalence of sarcopenia has been estimated to be 10% [3]. In Iran, more than 16% of elderly people are affected [4]. Sarcopenia results in a poor quality of life [5, 6] and it imposes a large economic burden to patients and health care system [7].

Although the etiology is not clearly known, decreased levels of growth hormone, estrogen and testosterone and increased levels of cortisol, elevated pro-inflammatory cytokines and oxidative stress may play key role in its incidence [5, 8, 9]. Recent studies have shown that accelerated reactive oxygen species (ROS) leads to neuromuscular dysfunction and muscle fibers death [10, 11]. Diet can influence oxidative balance in the body. Previous studies have shown that adequate intake of dietary antioxidants, including carotenoids [12], vitamin C and D [13], vitamin E [14], selenium [13] and fruit and vegetables [15] can prevent and ameliorate sarcopenia and improve muscle strength by eliminating ROSs. Lauretani et al. showed that low level of blood selenium in elderly people was associated with poor skeletal muscle strength [16]. Dietary vitamin C and carotenoids was associated with fat free mass and lean body mass respectively in a large sample of woman in another cross-sectional study [17]. Summarized data in a systematic review and meta-analysis revealed that consuming antioxidant rich foods could better the outcome of sarcopenia in old-young adult ≥ 55 years old [18]. Results of pooled effect size of 3 randomized clinical trials showed that the intervention of higher fruit and vegetables consumption or antioxidant (magnesium, vitamin E and vitamin D) supplementation was associated with reduced time of getting up from a chair without armrests five times with the arms against the chest [18]. In contrast, some other studies failed to find any significant association between antioxidant intake and sarcopenia [19, 20]. For instance, Mediterranean dietary pattern, rich in antioxidants, was not related to sarcopenia [21]. In addition dietary vitamin E consumption was not related to lean body mass in another study [17]. Dietary total antioxidant capacity (TAC) is used as a tool for measuring the antioxidant capacity of the whole diet. Most previous investigations on the linkage between antioxidants and sarcopenia have considered individual antioxidants and data on the association between total dietary antioxidant capacity (d-TAC) and sarcopenia are lacking. As d-TAC considers the interactions and synergic antioxidant activities in total diet [22, 23], it is much more informative than individual antioxidants. Despite earlier controversies on the association of individual dietary antioxidants and risk of sarcopenia, we hypothesized that d-TAC is protectively associated with the risk through lowering free radicals in the body. Therefore, this study aimed to investigate the relationship between d-TAC and sarcopenia in Iranian elderly people.

## Method

### Participants

We conducted a population-based cross-sectional study from May to October 2011 in Tehran, Iran. The details of the study have been published previously [24]. We enrolled 300 elderly people (150 men and 150 women) aged ≥ 55 years from district 6 of Tehran using cluster random sampling method. We selected the head of each 30 cluster based on a ten-digit postal code and enrolled individuals aged ≥ 55 years, with the ability to move without crutches, walker or assistive devices and those without any active cancers (based on self-reported data). We did not include people who were susceptible to sarcopenia including individuals with artificial limbs or limb prosthesis and those with a history of debilitating disease including Congestive Heart Failure (CHF), Chronic Obstructive Pulmonary Disorder (COPD), Chronic Renal Failure (CRF), cirrhosis and liver failure (based on self-reported data) [25].

The study protocol was approved by the Tehran University of Medical Sciences ethics committee. At first, participants were briefly informed about the objectives of the survey. All participants completed the written informed consent before data collection. Then all required data were collected through face-to-face method by a trained interviewer at home.

### Dietary assessments

Dietary intake of study participants were examined by the use of a Block-format 117-item food frequency questionnaire (FFQ) [26]. The questionnaire included a list of food items, along with a given portion size and an open-ended frequency response section. A trained nutritionist administered the FFQs. Participants were requested to report their daily, weekly or monthly frequency consumption of each food item in the questionnaire during the preceding year. Frequency data in the FFQ were then converted to grams per day considering the household measures of portion sizes. Then these data were linked to the modified food composition database of the US Department of Agriculture, using Nutritionist IV software [27], to compute daily energy and nutrients intake of each participant.

Although the FFQ was validated previously, we conducted a pilot study on 30 participants to examine its validity in elderly population. To do this, nutrient intakes of the FFQ were compared with those obtained from four dietary records (two records in weekdays and two other records after 2 months). The results showed a good correlation between the dietary intakes determined by the FFQ and those from the self-reported records. The correlation coefficient for animal protein, fruits, and vegetables were 0.43, 0.57, and 0.45, respectively. The energy adjusted correlation coefficients for β-carotene and vitamin C were 0.65 and 0.76, respectively.

### Calculation of dietary total antioxidant capacity

In this study, dietary total antioxidants capacity was computed using ferric reducing antioxidant power (FRAP). Data on TAC of foods was gathered from published databases that provided the antioxidant capacity measured by FRAP [28]. If TAC data were not available for any food item, the value of the nearest comparable food was assigned. Intake of each food item was converted to grams consumed per day and total antioxidant capacity intake was calculated by summing the product of grams consumed over all food items and units of antioxidant index per gram from an antioxidant index database.

### Assessment of sarcopenia

Sarcopenia was defined based on European Working Group on Sarcopenia (EWGSOP) definition [25]. EWGSOP recommends considering the combination of both low muscle mass and low muscle function (either strength or performance) in the definition. The muscle mass was measured as the ratio of an individual’s total lean mass of legs and arms (also named Appendicular Skeletal Muscle or ASM) [29] to their squared height (ASM/height²). A DXA scanner (Discovery W S/N 84,430) was used to measure ASM. According to EWGSOP, low muscle mass was defined as the amount of less than 5.45 (kg/m²) and 7.26 (kg/m²) for women and men, respectively [25]. A hand grip test by a pneumatic instrument -a squeeze bulb dynamometer (c7489-02 Rolyan) calibrated in pound per square inch (psi)- was used to scale the muscle strength. The hand grip strength (maximum voluntary contractions) was measured three times for each right and left hand with a 30-second rest in between measurements. We used the average measurements of the participants’ both hands as their muscle strength. Sex and age-specific cutoff points suggested by Merkies et al. were used to identify low muscle strength [29]. The muscle performance was measured using a 4-Meter walk gait speed test [30]. Participants who had gait speeds less than 0.8 m/s were identified as those with a low muscle performance [25].

### Assessment of other variables

General information on age, sex, socio-economic status, marital status, education, medical history, medication use, smoking habits and alcohol consumption was collected by a trained dietitian. Physical activity level was examined by the use of a short form of International Physical Activity Questionnaire (IPAQ). The validity of IPAQ has previously been examined in elderly population [31]. Vigorous- and moderate-intensity activities and walking (for at least 10 min) were asked separately in minutes and days during the past week. Then MET scores for each activity were obtained from earlier publications [32]. The obtained MET scores were multiplied by the amount of time each participant spent on that activity, while taking into consideration the frequency of engaging in the mentioned activity during the past week. Then, the scores for different activities were summed up to obtain total MET-mins/week. Weight was measured using a digital scale while participants were minimally clothed. A wall tape measure was used to assess height in standing position without shoes. Participants were asked to stand up and normally breathe to measure waist circumference at the middle of lower rib margin and iliac crest. Weight (kg) divided by height squared (m^2^) was used to calculate body mass index (BMI).

### Statistical analysis

In this study, participants were categorized based on tertiles of d-TAC. We did ANOVA for comparing means of continuous variables and chi-square test for investigating the distribution of categorical variables across tertiles of d-TAC. To compere dietary nutrients and food groups’ intakes across tertiles of d-TAC, we applied ANCOVA which was adjusted for age, sex and energy intake. Means of muscle mass, hand grip strength and gait speed, as components of sarcopenia, were compared across tertiles of d-TAC using ANOVA. To examine the association between d-TAC and sarcopenia we used binary logistic regression in three different models. First, we controlled for age, sex and energy intake. Further adjustment was performed for physical activity, marital status, smoking, alcohol use, medication use (statin, ACEi, estrogen, testosterone), and history of disease (asthma, arthritis, MI, CVA). We also controlled for BMI in the last model. The first tertile of d-TAC was defined as the reference category and odds ratios and 95% CIs in the second and third tertiles were computed. The overall trend of ORs across increasing tertiles of d-TAC was evaluated by defining tertiles of d-TAC as ordinal variable. Further we applied subgroup analysis as supplementary analysis bases on sex to explore sex-associate differences. SPSS (SPSS Inc., version 22) was used for all the analyses. P values < 0·05 were defined as significant.

## Results

Baseline characteristics of study participants across tertiles of d-TAC are provided in Table 1. Participants in the top tertile of d-TAC were physically active, more likely to be men, alcohol user and smoker than those in the bottom tertile. No other significant difference was seen in terms of mean age, BMI and waist circumferences as well as in the distribution of participants in terms of marital status, education, history of diabetes, MI, asthma and medication use across tertile categories of d-TAC.

**Table 1:** Baseline Characteristics of study participants across tertiles of dietary TAC

	Tertiles of dietary TAC			P^b^
	T1 (< 10.82)	T2 (10.82–14.59 )	T3 ( 14.59< )	
Age (years)^a^	67.5 ± 8.1	67.07 ± 7.66	65.58 ± 7.17	0.16
BMI (kg/m2) ^a^	27.3 ± 4.16	27.4 ± 4.59	27.39 ± 3.93	0.96
Waist Circumference (cm) ^a^	96.4 ± 10.1	96.3 ± 10.9	97.1 ± 8.93	0.81
Physical activity(Met-h/week) ^a^	13.4 ± 1.34	26.6 ± 2.67	25.95 ± 2.7	0.007
Male (%)	40.4	46.5	58.6	0.03
Married (%)	79.8	76.8	80.8	0.76
Alcohol use^c^ (%)	9.1	9.1	21.2	0.01
Smoking^d^ (%)	8.1	9.1	20.2	0.01
Education (Above diploma) (%)	34.3	32.7	47.4	0.06
History of diabetes (%)	19.2	16.2	27.3	0.13
History of MI (%)	10.1	9.1	16.2	0.24
History of Asthma (%)	2	2	2	0.99
History of Sexual hormone use (%)	4	2	3	0.70
History of Statin use (%)	40.4	28.3	40.4	0.12
History of Corticosteroid use (%)	2	4	2	0.59

MI, Myocardial infarction; CVA, Cerebrovascular accident

^a^Data are mean ± SD.

^b^ Obtained from ANOVA or chi-square test, where appropriate

^c^ History of alcohol use in the past 6 month

^d^ History of smoking use in the past 1 month

Higher d-TAC was associated with higher intake of energy, total dietary fiber, riboflavin, vitamin C, folic acid as well as fruits and vegetables. There was no significant difference in dietary intake of carbohydrates, proteins, fats, vitamins E and B6, selenium, iron, refined grains, whole grains, red meat, processed meats, low-fat dairy, high-fat dairy and nuts and legumes across tertiles of d-TAC (Table 2**)**.

**Table 2:** Dietary intakes of participants by tertiles of dietary TAC^1^

	Tertiles of dietary TAC			P
	T1 (< 10.82 )	T2 (10.82–14.59 )	T3 (14.59<)	
**Nutrients**				
Energy intake (kcal/d)	1802 ± 84.9	2233 ± 84.4	2741 ± 85.3	< 0.001
Carbohydrate (g/d)	369 ± 5.81	365 ± 5.5	360 ± 5.8	0.61
Protein g/d)	83.5 ± 1.90	87.1 ± 1.80	87.4 ± 1.92	0.29
Fat (g/d)	57.8 ± 1.99	58.2 ± 1.89	61.33 ± 2.01	0.43
Total dietary fiber (g/d)	27.7 ± 0.88	29.5 ± 0.83	32.60 ± 0.88	0.001
Vitamin E (mg/d)	8.74 ± 0.53	9.52 ± 0.50	9.95 ± 0.53	0.29
Vitamin B6 (mg/d)	2.57 ± 0.13	2.56 ± 0.12	2.69 ± 0.13	0.76
Riboflavin (mg/d)	2.18 ± 0.05	2.46 ± 0.05	2.51 ± 0.05	< 0.001
Vitamin C (mg/d)	224 ± 10.4	282 ± 9.89	316 ± 10.5	< 0.001
Selenium (µg/d)	0.09 ± 0.004	0.10 ± 0.003	0.09 ± 0.004	0.67
Iron (mg/d)	19.5 ± 0.36	19.8 ± 0.34	20.6 ± 0.36	0.10
Folic acid (mg/d)	484 ± 11.3	550 ± 10.7	594 ± 11.4	< 0.001
**Food Groups**				
Fruit (g/d)	474 ± 192	670 ± 254	802 ± 323	< 0.001
Vegetables (g/d)	459 ± 198	565 ± 247	678 ± 309	< 0.001
Refined grains (g/d)	232 ± 156	255 ± 186	274 ± 237	0.06
Whole grains (g/d) (g/d)	47.4 ± 50.6	57.4 ± 62.9	79.9 ± 93.5	0.46
Red meat (g/d)	29.6 ± 27.9	33.8 ± 26.3	42.5 ± 37.3	0.91
Processed meats (g/d)	2.26 ± 4.54	1.35 ± 3.61	3.11 ± 11.01	0.36
Low-fat dairy (g/d)	114 ± 136	171 ± 218	162 ± 171	0.056
High-fat dairy (g/d)	335 ± 203	415 ± 263	448 ± 331	0.64
Nuts and legumes (g/d)	41.7 ± 32.2	52.6 ± 36.3	62.1 ± 47.8	0.84

All values were adjusted for age, sex and energy, except for dietary energy intake, which was only adjusted for age and sex using ANCOVA.

Table 3 indicates means of muscle mass, hand grip strength and gait speed as well as prevalence of components of sarcopenia across tertiles of d-TAC. Individuals in the highest tertile of di-TAC had greatest hand grip strength (11.9 vs. 10.4 psi *P* = 0.009) and tended to have higher mean muscle mass than those in the lowest tertile (6.73 vs. 6.41 kg, *P* = 0.06). Although, the prevalence of components of sarcopenia was not significantly different across tertile categories of d-TAC, we found that individuals in the top category of dietary TAC had significantly lower prevalence of sarcopenia than those in the bottom category (10.1 vs. 22.2%, *P* = 0.04).

**Table 3:** Components of sarcopenia across tertiles of dietary TAC

	Tertiles of dietary TAC			P^a^
	T1 (< 10.82 )	T2 (10.82–14.59 )	T3 (14.59<)	
Muscle mass (kg)^b^	6.41 ± 0.98	6.65 ± 1.05	6.73 ± 0.91	0.06
Hand grip strength (psi) ^b^	10.4 ± 3.55	10.7 ± 3.34	11.9 ± 3.63	0.009
Gait speed (m/s) ^b^	0.82 ± 0.22	0.85 ± 0.23	0.85 ± 0.21	0.46
Abnormal Muscle mass (%) ^c^	42.4	36.4	38.4	0.67
Abnormal Hand grip strength (%) ^d^	32.3	38.4	26.3	0.19
Abnormal Gait speed (m/s) (%) ^e^	45.5	40.4	35.4	0.35
Sarcopenia (%)	22.2	21.2	10.1	0.04

^a^ Obtained from ANOVA or chi-square test, where appropriate

^b^ Data are mean ± SD.

^c^ Muscle mass lower than 5.45 (kg/m²) for women and 7.26 (kg/m²) for men were considered abnormal

^d^ Abnormal muscle strength was defined according previous study [34]

^e^ Gait speeds lower than 0.8 m/s were considered abnormal

Multivariable-adjusted ORs and 95% CIs for sarcopenia across tertiles of d-TAC are presented in Tables 4, 5. Individuals in the highest tertile of d-TAC had 61% (95% CI: 0.17, 0.88) lower odds of sarcopenia compared with those in the lowest tertile. After adjustment for age, sex and energy intake, the association strengthened; such that participants with the greatest TAC were 74% (95% CI: 0.10, 0.67) less likely to have sarcopenia compared with those with the lowest d-TAC. Further adjustment for other potential confounders did not alter the association (OR = 0.29; 95% CI: 0.11, 0.77). Even after additional controlling for BMI, individuals in the highest tertile of dietary TAC had lower odds of sarcopenia than those in the lowest tertile (OR = 0.33; 95% CI: 0.11, 0.95). However, when we examined the association between d-TAC and components of sarcopenia, no significant association was seen either before or after adjustment for confounders.

**Table 4:** Multivariate adjusted odds ratio for sarcopenia and its components across tertiles of dietary TAC

		Tertiles of dietary TAC			P-trend
		T1 (< 10.82 )	T2 (10.82–14.59 )	T3 (14.59<)	
		OR	OR (95% CI)	OR (95% CI)	
Sarcopenia					
Crude		1	0.94 (0.47, 1.85)	0.39 (0.17, 0.88)	0.02
Model 1		1	0.81 (0.40, 1.63)	0.26 (0.10, 0.67)	0.007
Model 2		1	0.86 (0.41, 1.48)	0.29 (0.11, 0.77)	0.009
Model 3		1	1.007 (0.43, 2.31)	0.33 (0.11, 0.95)	0.050
Abnormal muscle mass					
Crude		1	0.77 (0.43, 1.37)	0.84 (0.47, 1.49)	0.56
Model 1		1	0.68 (0.36, 1.27)	0.65 (0.33, 1.29)	0.22
Model 2		1	0.66 (0.34, 1.26)	0.57 (0.28, 1.19)	0.13
Model 3		1	0.59 (0.26, 1.37)	0.71 (0.29, 1.78)	0.46
Abnormal hand grip strength					
Crude	1		1.30 (0.72, 2.34)	0.74 (0.40, 1.37)	0.36
Model 1	1		1.37 (0.72, 2.59)	0.78 (0.37, 1.62)	0.54
Model 2	1		1.39 (0.73,2.65)	0.88 (0.42, 1.88)	0.82
Model 3	1		1.40 (0.73, 2.68)	0.90 (0.42, 1.91)	0.85
Abnormal gait speed					
Crude	1		0.81 (0.46, 1.42)	0.65 (0.37, 1.16)	0.14
Model 1	1		0.88 (0.48, 1.62)	0.87 (0.44, 1.70)	0.68
Model 2	1		0.96 (0.51, 1.79)	0.99 (0.48, 2.01)	0.98
Model 3	1		0.95 (0.51, 1.80)	1.007 (0.49, 2.05)	0.99

TAC, Total antioxidant capacity

Data are OR (95% CI)

Model 1: adjusted for age, sex and energy intake

Model 2: further adjustments were made for physical activity, education, marital status, smoking, alcohol use, medication use (statin, ACEi, estrogen, testosterone), and history of disease (asthma, arthritis, MI, CVA)

Model 3: additionally adjusted for BMI.

**Table 5:** Multivariate adjusted odds ratio for sarcopenia and its components across tertiles of dietary TAC based on sex

		Tertiles of dietary TAC			P-trend
		T1 (< 10.82 )	T2 (10.82–14.59 )	T3 (14.59<)	
		OR	OR (95% CI)	OR (95% CI)	
**Sarcopenia**					
**Male**					
Crude		1	1.039 (0.40, 2.67)	0.36 (0.12, 1.03)	0.05
Adjusted model		1	2.34 (0.70, 7.76)	0.70 (0.18, 2.68)	0.58
**Female**					
Crude		1	0.77 (0.28, 2.10)	0.34 (0.90, 1.32)	0.62
Adjusted model		1	0.38 (0.92, 1.58)	0.11 (0.13, 1.05)	0.04
**Abnormal muscle mass**					
**Male**					
Crude		1	0.53 (0.22, 1.25)	0.73 (0.32, 1.64)	0.53
Adjusted model		1	0.69 (0.22, 2.13)	0.90 (0.28, 2.88)	0.91
**Female**					
Crude		1	0.98 (0.44, 2.20)	0.64 (0.25, 1.61)	0.37
Adjusted model		1	0.92 (0.24, 3.44)	0.97 (0.18, 5.19)	0.96
**Abnormal hand grip strength**					
**Male**					
Crude	1		2.06 (0.73, 5.77)	0.34 (0.09, 1.28)	0.10
Adjusted model	1		3.14 (0.92, 10.69)	0.36 (0.80, 1.62)	0.64
**Female**					
Crude	1		1.12 (0.53, 2.37)	1.57 (0.70, 3.51)	0.27
Adjusted model	1		0.99 (0.45, 2.19)	1.28 (0.49, 3.28)	0.63
**Abnormal gait speed**					
**Male**					
Crude	1		0.53 (0.21, 1.30)	0.43 (0.18, 1.02)	0.06
Adjusted model	1		0.63 (0.22, 1.77)	0.75 (0.26, 2.14)	0.61
**Female**					
Crude	1		1.15 (0.54, 2.41)	1.16 (0.52, 2.58)	0.61
Adjusted model	1		1.11 (0.45, 2.73)	1.04 (0.33, 3.21)	0.92

TAC, Total antioxidant capacity

Data are OR (95% CI)

Adjusted model: adjusted for age, sex, energy intake, physical activity, education, marital status, smoking, alcohol use, medication use (statin, ACEi, estrogen, testosterone), and history of disease (asthma, arthritis, MI, CVA) and BMI.

We also obtained multivariable-adjusted ORs and 95% CIs for sarcopenia across tertiles of dietary TAC based on sex (**supplementary table**). No significant association was seen between dietary TAC and sarcopenia and its components in male and female separately.

## Discussion

In this cross-sectional study, we found an inverse association between dietary total antioxidant capacity and odds of sarcopenia. This association persisted in multivariate models accounting for potential confounders. In addition, people in the highest tertile of d-TAC had the greatest hand grip strength. To the best of our knowledge, this is the first study examining the association between d-TAC and sarcopenia.

Sarcopenia is known to contribute to metabolic disorders, morbidity and mortality from chronic diseases [33, 34]. In the present study, after adjustment for potential confounders, individuals with the greatest dietary TAC had 67% decreased risk of sarcopenia. There are increasing interests in the role of oxidative stress in the etiology of sarcopenia. We did not find any earlier study in the literature to compare our findings with; however, the whole dietary patterns as well as individual dietary antioxidants had been assessed in relation to sarcopenia. Fruit and vegetables as well as the Mediterranean dietary pattern seem to provide high amounts of dietary antioxidants. Prior studies had shown that dietary intake of fruits was associated with > 20% reduced risk of sarcopenia in Korean elderly people. The same association was also reported for vegetables [15]. Men with a higher adherence to “vegetables-fruits dietary pattern” had lower odds of sarcopenia [35]. Findings from another study revealed that women with the greatest adherence to the Mediterranean diet lost less relative skeletal muscle index (RSMI) and total body lean mass as compared with those with the lowest adherence [36]. Unlike these findings, a study in Chinese people showed that the adherence to the Mediterranean dietary pattern was not associated with odds of sarcopenia [35]. Dietary intakes of individual antioxidants including vitamins A, E, C and selenium was not also significantly different between class Ι sarcopenic Canadian adults and healthy ones [20]. Given the interaction among nutrients as well as their synergistic effects on each other, the effect of total antioxidant capacity might be different from the effect of individual antioxidants. Therefore, assessment of the effect of individual antioxidants in the diet might not reflect the whole antioxidant capacity of the diet [37]. It must be noted that studies in which no association was seen between dietary antioxidants intake and sarcopenia had some differences with our study. For example, they had smaller sample size than our study [20], used different method to evaluate dietary intakes [20], had made no adjustment for covariates [20] or had lower than usual adherence to Mediterranean diet [35].

We failed to find any significant association between d-TAC and components of sarcopenia including muscle mass, hand grip strength and gait speed. In line with our study, some other investigators study the same findings. For instance, adherence to the Mediterranean diet was not associated with grip strength in postmenopausal elderly Finnish women [36]. In contrast, consumption of prudent diet, which was characterized by high consumption of fruit, vegetables, whole-grain cereals, and fatty fish was associated with greater grip strength in the United Kingdom [38]. In another study, low adherence to the Mediterranean diet was associated with lower lean mass and lower walking speed [36]. It must be kept in mind that number of people with different components of sarcopenia was low in our study. Therefore, this study might be underpowered to find such a relationship.

The mechanisms through which dietary antioxidants might affect the risk of sarcopenia, remain unknown. It seems that oxidative stress is the central mechanism implicated in the pathogenesis of sarcopenia. Skeletal muscle is the greatest consumer of oxygen in the body. Type Ι muscle fiber has oxidative metabolism and continuously generate ROS in the body. As muscles get older, oxidative harms resulted in a conversion of type ΙΙ muscle fiber with more myosin heavy chains to type Ι fiber with less myosin heavy chains. Therefore, power, speed and the capacity of normal activity of muscle will reduce [39–41]. Documents also have demonstrated that biomarkers of oxidative damage are raised in older adults [41]. This oxidative damage might affect DNA, protein, and lipids. Antioxidants can influence the enzymatic profile of body defense including superoxide dismutase (SOD) and glutathione peroxidase (GP); enzymes that reduce ROS. Moreover, they modulate redox-sensitive transcription factors such as NF-κB, which are involved in the up-regulation of pro-inflammatory cytokines, which in turn contribute to sarcopenia [41–44]. In the other hand, the function of antioxidant system also is affected by age which the occurred reduction will worst the condition.

Our study had several strengths. To the best of our knowledge, this is the first study examining the association between d-TAC and sarcopenia. In addition, we controlled for several confounding variables to reach to an independent association between d-TAC and sarcopenia. Moreover, we examined sarcopenia using DEXA, which is the gold standard method for the diagnosis and characterization of sarcopenia [45]. We did not restrict the analysis to patients with sarcopenia and tried our best to examine the associations between d-TAC and components of sarcopenia (including muscle mass, hand grip strength and gait speed) as well. Finally, we used a validated FFQ to evaluate dietary intakes of study participants. Nevertheless, some limitations must be considered. First, cross-sectional studies has their own methodological limitations [46]. For example the causality in these studies cannot be inferred. Furthermore, as exposure and outcome are measured in the same time, individuals with sarcopenia may have been altered their diets in an effort to improve their muscle performance. In addition, although we controlled the analysis for several potential confounders, residual confounding cannot be excluded. Another limitation of our study was its small sample size. However it was still much more than some previous published studies which investigated the association between diet and sarcopenia [47, 48]. Using well-known formula for measuring sample size in cross-sectional studies [49] and considering type one error (α) = 0.05, we reached to 244 participant in which the sample size of the current study met this number. We also cannot rely on subgroup analysis results due to data fragmentation and minifying the sample size. It must also be acknowledged that the study sample was not a representative sample of Iranian population; therefore, the generalizability of our findings should be done cautiously. As with all epidemiologic studies, the use of FFQ for dietary assessment is always associated with some errors in classification of study participants in terms of dietary exposure [50]. However, we used a valid and reliable FFQ that might help reducing this type of error.

## Conclusion

In conclusion, based on our results, higher intake of dietary antioxidants could inversely associated with sarcopenia. Therefore encouraging the elderly people to receive more dietary antioxidants such as fruits (especially red fruits) and dried fruits, vegetables, olive, coffee and nuts can be an affordable solution for nutritional and beneficial interventions in preventing sarcopenia and maintaining muscle health. No significant association was seen between d-TAC and individual components of sarcopenia. Further studies are needed to confirm our findings.

### Electronic supplementary material

Below is the link to the electronic supplementary material.

Supplementary Material 1

## Data Availability

The datasets used and analyzed during the current study are available from the corresponding author on reasonable request.

## Abbreviations

d-TAC: Dietary Total Antioxidant Capacity
EWGSOP: European Working Group on Sarcopenia
DXA: Dual-energy X-ray absorptiometry
ASM: Appendicular Skeletal Muscle
FFQ: Food Frequency Questionnaire
ROS: Reactive Oxygen Species. FRAP:Ferric Reducing Antioxidant Power
IPAQ: International Physical Activity Questionnaire
MET: Metabolic equivalents
BMI: Body Mass Index
ANCOVA: Analysis of Covariance
MI: Myocardial Infraction
RSM: Relative Skeletal Muscle Index
