# Supplementary material for: Associations between dietary total antioxidant capacity and sarcopenia: a cross-sectional study
Source: Nutr J. 2024 Jul 31;23:87. doi: 10.1186/s12937-024-00933-5 (PMC11290090; doi:10.1186/s12937-024-00933-5)
Supplement: Supplementary file 1 — Supplementary Material 1 [file 12937_2024_933_MOESM1_ESM.docx]

**Supplementary table:** Multivariate adjusted odds ratio for sarcopenia and its components across tertiles of dietary TAC based on sex.

|  | | Tertiles of dietary TAC | | | P-trend |
| --- | --- | --- | --- | --- | --- |
|  |  | T1  (<10.82 ) | T2  (10.82 -14.59 ) | T3  (14.59<) |  |
|  |  | OR | OR (95% CI) | OR (95% CI) |  |
| **Sarcopenia** | |  |  |  |  |
| **Male** | |  |  |  |  |
| Crude | | 1 | 1.03 (0.40, 2.67) | 0.36 (0.12, 1.03) | 0.05 |
| Adjusted model | | 1 | 2.34 (0.70, 7.76) | 0.70 (0.18, 2.68) | 0.58 |
| **Female** | |  |  |  |  |
| Crude | | 1 | 0.77 (0.28, 2.10) | 0.34 (0.90, 1.32) | 0.62 |
| Adjusted model | | 1 | 0.38 (0.92, 1.58) | 0.11 (0.13, 1.05) | 0.04 |
| **Abnormal muscle mass** | | |  |  |  |
| **Male** | |  |  |  |  |
| Crude | | 1 | 0.53 (0.22, 1.25) | 0.73 (0.32, 1.64) | 0.53 |
| Adjusted model | | 1 | 0.69 (0.22, 2.13) | 0.90 (0.28, 2.88) | 0.91 |
| **Female** | |  |  |  |  |
| Crude | | 1 | 0.98 (0.44, 2.20) | 0.64 (0.25, 1.61) | 0.37 |
| Adjusted model | | 1 | 0.92 (0.24, 3.44) | 0.97 (0.18, 5.19) | 0.96 |
| **Abnormal hand grip strength** | | |  |  |  |
| **Male** |  | |  |  |  |
| Crude | 1 | | 2.06 (0.73, 5.77) | 0.34 (0.09, 1.28) | 0.10 |
| Adjusted model | 1 | | 3.14 (0.92, 10.69) | 0.36 (0.80, 1.62) | 0.64 |
| **Female** |  | |  |  |  |
| Crude | 1 | | 1.12 (0.53, 2.37) | 1.57 (0.70, 3.51) | 0.27 |
| Adjusted model | 1 | | 0.99 (0.45, 2.19) | 1.28 (0.49, 3.28) | 0.63 |
| **Abnormal gait speed** | | |  |  |  |
| **Male** |  | |  |  |  |
| Crude | 1 | | 0.53 (0.21, 1.30) | 0.43 (0.18, 1.02) | 0.06 |
| Adjusted model | 1 | | 0.63 (0.22, 1.77) | 0.75 (0.26, 2.14) | 0.61 |
| **Female** |  | |  |  |  |
| Crude | 1 | | 1.15 (0.54, 2.41) | 1.16 (0.52, 2.58) | 0.61 |
| Adjusted model | 1 | | 1.11 (0.45, 2.73) | 1.04 (0.33, 3.21) | 0.92 |

TAC, Total antioxidant capacity

Data are OR (95% CI)

Adjusted model: adjusted for age, sex, energy intake, physical activity, education, marital status, smoking, alcohol use, medication use (statin, ACEi, estrogen, testosterone), and history of disease (asthma, arthritis, MI, CVA) and BMI.
